# Development of *in situ* bioprinting: A mini review

**DOI:** 10.3389/fbioe.2022.940896

**Published:** 2022-07-22

**Authors:** Aidan MacAdam, Emaan Chaudry, Christopher D. McTiernan, David Cortes, Erik J. Suuronen, Emilio I. Alarcon

**Affiliations:** ^1^ BEaTS Research, Division of Cardiac Surgery, University of Ottawa Heart Institute, Ottawa, ON, Canada; ^2^ Faculty of Medicine, University of Ottawa, Ottawa, ON, Canada; ^3^ Department of Cellular and Molecular Medicine, Faculty of Medicine, University of Ottawa, Ottawa, ON, Canada; ^4^ Department of Biochemistry, Microbiology and Immunology, Faculty of Medicine, University of Ottawa, Ottawa, ON, Canada

**Keywords:** *in situ* bioprinting, handheld bioprinter, bedside mounted bioprinter, minimally invasive, crosslinking, bioink

## Abstract

Bioprinting has rapidly progressed over the past decade. One branch of bioprinting known as *in situ* bioprinting has benefitted considerably from innovations in biofabrication. Unlike *ex situ* bioprinting, *in situ* bioprinting allows for biomaterials to be printed directly into or onto the target tissue/organ, eliminating the need to transfer pre-made three-dimensional constructs. In this mini-review, recent progress on *in situ* bioprinting, including bioink composition, *in situ* crosslinking strategies, and bioprinter functionality are examined. Future directions of *in situ* bioprinting are also discussed including the use of minimally invasive bioprinters to print tissues within the body.

## Introduction

Advancements made in the technology and design of three-dimensional (3D) bioprinting has allowed for tissue engineering research to progress rapidly (Li et al., 2016). Bioprinting is a form of 3D printing where bioinks/biomaterial inks are used to print structures that mimic native tissues. Developing 3D constructs has allowed for implants to be tailored and designed for individual patient anatomy. Recently, there have been several advancements regarding *in situ* 3D bioprinting. *In situ*, meaning “on the spot,” has allowed for the level of personalization of these therapies to be taken a step further. During *in situ* bioprinting, the construct is printed directly onto or into the damaged tissue or organ. One of the main disadvantages of *ex situ* bioprinting is the need to transport pre-made constructs from the printer for application to the tissue or organ. Additionally, it increases the risk of introducing an infection to the material that can be passed on to the host (Elemoso et al., 2020). The immediate transfer of biomaterials to the patient during the *in situ* bioprinting process eliminates many of the associated issues of *ex vivo* bioprinting. *In situ* bioprinted constructs may also demonstrate improved functionality and integration compared to *ex situ* printed implants due to benefits incurred from the natural cellular microenvironment of the body (Murdock and Badylak, 2017). However, the extent of biological improvement of *in situ* bioprinted grafts remains unknown. In effect, future research that shows *in situ* bioprinted grafts perform better statistically than preprinted constructs would only help accelerate the development of *in situ* bioprinting technology.

**FIGURE 1:**
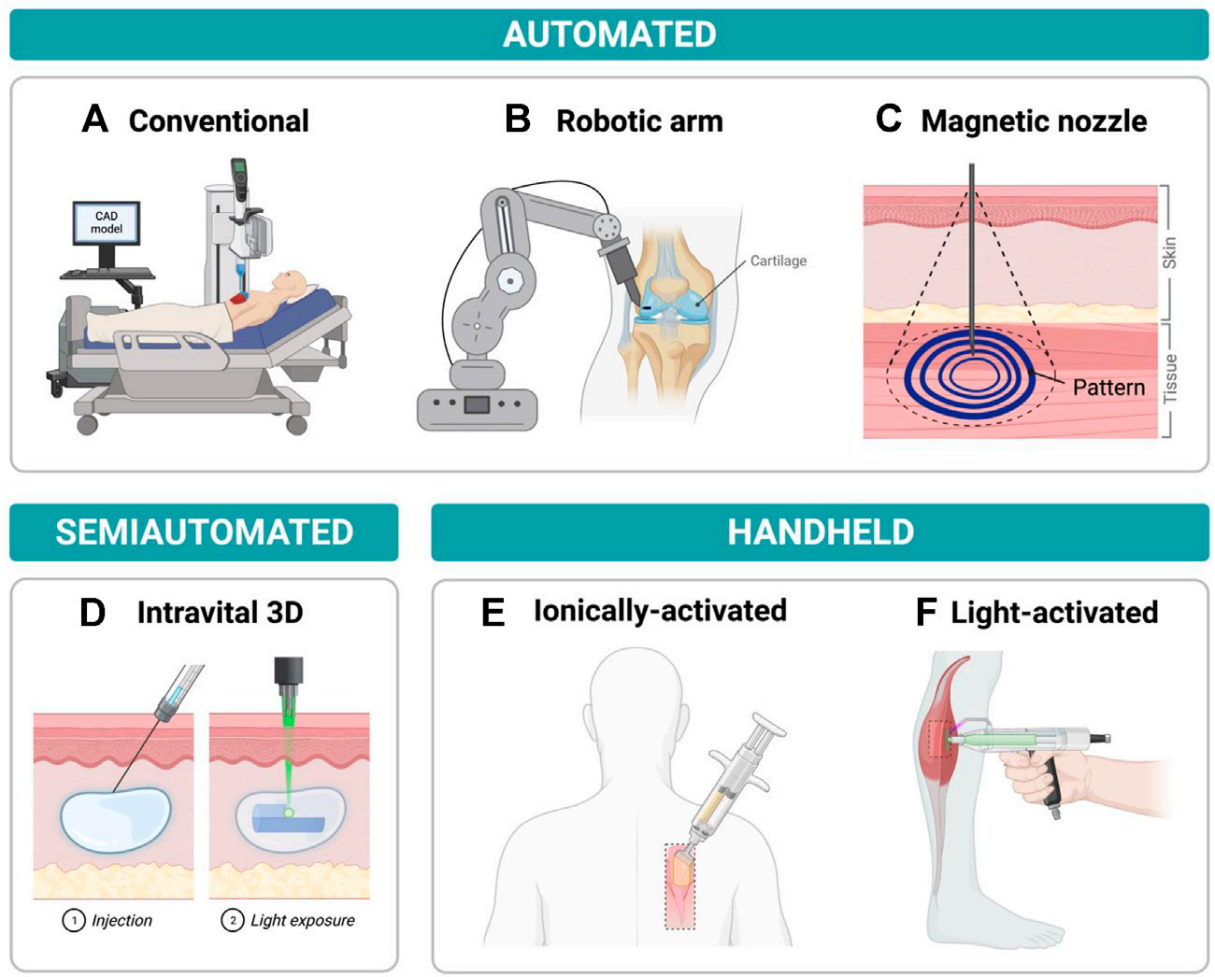
Examples of current *in situ* bioprinters with a focus on printers that show potential in minimally invasive repair **(A)** Traditional *in situ* bioprinter moving along *x*-*y*-*z* axes while depositing bioink onto chest wound according to a computer-aided design (CAD) model **(B)** Robotic arm-assisted bioprinter delivering bioink to cartilage injury from an advantageous position due to the high rotational freedom of the robot. **(C)** Magnetoactive soft nozzle printing a circular pattern onto tissue beneath the skin’s surface through magnetic actuation. **(D)** Photocrosslinking light-sensitive biopolymers that have been injected into the dermis by intravital 3D (i3D) bioprinting to form a final hydrogel structure. **(E)** Printing sheets of biomaterials onto dorsal full thickness skin wound by delivering hydrogel precursor solution and crosslinker solution concurrently to wound site using a handheld printer. **(F)** Delivering photopolymerizable bioink to muscle injury and crosslinking bioink with blue/purple light using a handheld device.

There are various types of bioprinters and printing techniques that can be used to aid in the reconstruction and regeneration of damaged tissue. In general, *in situ* bioprinters can be organized into two groups: bedside mounted printers and hand-held printers. Herein, bedside mounted bioprinters will be defined as printers that can fit around the subject and print directly onto the area of interest. Hand-held printers meanwhile are small devices that can be manually operated allowing for increased flexibility and surgical dexterity, see Figure 1.

The *in situ* bioprinting field has progressed rapidly in the last few years, and while several review articles have been published regarding bioprinting in general, few review articles have focused on the development of *in situ* bioprinting (Agarwal et al., 2020; Saini et al., 2021; Weng et al., 2021). As a result, some key advantages/disadvantages associated with different *in situ* bioprinting techniques may have been overlooked. In this mini review, advances *in situ* bioprinting will be discussed including the recent development of minimally invasive technologies. The progression of bedside mounted and handheld bioprinting devices are also discussed in detail and many of the key features of these bioprinters are summarized in Tables 1 and 2. This review will also look at the performance of different bioinks used to 3D print unique structures in various animal models.

**TABLE 1:** *In situ* 3D bioprinting—bedside mounted bioprinting.

Printer	Bioink(s)	Target tissues/Organs	Cell viability	*In Vivo* model	Goals and outcomes	Special features or properties	References
Custom-made bioprinter	Mesenchymal stem cells (MSCs) and amniotic fluid-derived stem (AFS) cells	Skin	The bioprinting procedure consistently yielded a fibrin/collagen gel that provided 100% coverage over the wound area and formed a tight seal with the skin at the edges of the excisional wound	Mouse	Results indicated that bioprinting with amniotic fluid-derived (AFS) cells could be an effective treatment for large-scale wounds and burns. AFS cells were used in the bioink due to their high proliferation capacity, multipotency, and immunomodulatory activity	Uses MSC’s and AFS cells in the bioink	Skardal et al. (2012)
Custom-made bioprinter (i3Dbioprinting)	3D cell-laden photosensitive polymer hydrogels	Skin, skeletal muscle and brain	Two days after 3D culture, cell viability was between 90% and −99% in all conditions	Mouse	The high compatibility of i3D bioprinting was confirmed by the integrity of the skin after the procedure. Photosensitive solutions were crosslinked in skeletal muscle without evident alteration of the overall muscle-fibre morphology and connective-tissue integrity	i3D bioprinting is performed by injection, fabrication of 3D hydrogel objects by two-photon excitation and intravital imaging for hydrogel identification and *in vivo* analysis	Urciuolo et al. (2020)
Custom-made bioprinter (HT-BioLP Workstation)	Nano-hydroxyapatite (n-HA)	Bone (calvarial defects)	After 1 month, newly formed mature and immature bones and n-HA aggregates inside macrophages were observed in test sites, while no bone repair was seen in control sites	Mouse	Nano-hydroxyapatite (n-HA) bioink was shown to be biocompatible with osteoblastic cells and caused no inflammation *in vivo*	A “first attempt to apply bioprinting technologies in the perspective of computer-assisted medical interventions.”	Keriquel et al. (2010)
Custom-made bioprinter	HAMA and 4-Armed PEG-ACLT were dissolved in phosphate-buffered saline (PBS) solution to a final concentration of 2% and 5% w/v	Knee joint (cartilage repair)	The control group did not fill the defect region. In the hydrogel implantation group and *in situ* bioprinting group, the defects were fully filled by newly-born tissue (hyaline cartilage)	Rabbit	The accuracy of the robot could be notably improved, and the error of the printed surface was less than 30 μm. The osteochondral defect could be repaired during about 60 s and compared with traditional ball-bar instruments, the fast TCP calibration demonstrated a noticeable improvement in measuring space and operation process	Introduction of the 6-DOF robot achieved a larger workspace and satisfied printing accuracy. The accuracy of bioprinting was easily ameliorated by the TCP calibration method. The whole procedure can be accomplished by non-professionals	Ma et al. (2020)
Ferromagnetic soft catheter robot (FSCR) bioprinter system	(1) viscoelastic bioink composed of Ecoflex and PDMS-1700. (2) conductive silver ink (3) conductive hydrogel ink	Liver	Hydrogel ink adhered to porcine tissue and maintained its shape after extrusion from the bioprinter. The FSCR nozzle which consisted of PDMS and hard-magnetic microparticles showed 98.6% cell viability suggesting high biocompatibility of the FSCR.	Rat	The FSCR bioprinting system was able to print multiple patterns on planar and curved surfaces with high accuracy. Bioprinting could be performed on internal organs through a minimally invasive surgery thanks to the compliant nozzle of the FSCR.	The FSCR bioprinter can print over a large area inside the body through a minor incision on the skin surface. Translational and rotational motion of the FSCR is achieved by four motor-driven permanent magnets	Zhou et al. (2021)

**TABLE 2:** *In situ* 3D bioprinting—hand-held bioprinting.

Printer	Bioink(s)	Target tissues/Organs	Cell viability	*In Vivo* model	Goals and outcomes	Special features or properties	References
Biopen	Core: HA-GelMa bioink (composed of gelatin methacrylamide (GelMa) and hyaluronic acid methacrylate (HAMA) hydrogel) + allogeneic adipose-derived MSCs Shell: HA-GelMa bioink + photoinitiator	Stifle (knee) joints	MSC viability was 97%. Histological assessment of repair showed no statistically significant difference when all groups were compared. The Handheld (HH) group showed a higher amount of newly regenerated cartilage. However, there was minimal lateral integration	Sheep	All animals underwent surgical 3D bioprinting without any intra- or postoperative complications. The Biopen allowed early cartilage regeneration	The device is manually operated, allows for surgical sculpting of tissue to achieve the users desired structure. It also allows for increased surgical dexterity and is a small, less cumbersome device that can easily be brought in/out of the surgical field	Di Bella et al. (2018)
Handheld skin printer	Bioinks of three compositions were used with dermal and epidermal cells embedded: 1. Alginate-collagen sheets, 2. Fibrin-based sheets, 3. Alginate sheets	Skin	The human dermal fibroblasts embedded in the fibrin sheets exhibited >90% viability. Sheets deposited in murine models remained firmly attached to the wound. In porcine models, 1 out of 4 control wounds showed complete re-epithelialization whereas 3 out of 4 treated wounds exhibited complete re-epithelialization	Mice and Pigs	The murine model was a first proof-of-concept experiment that demonstrated *in situ* deposition of an architected sheet in the form of a fiber array onto a small and compliant wound surface. The porcine model was a second proof-of-concept study that demonstrated the feasibility of using the handheld skin printer for *in situ* biopolymer sheet deposition	The printer is integrated, lightweight (<0.8 kg) and has a high degree of portability. It is straightforward to operate and can form biomaterial and tissue sheets with local control over the biomaterial composition	Hakimi et al. (2018)
Handheld instrument with microfluidic printhead	MSC-containing fibrin-HA bioink	Skin	Cultured cells in the 3D matrix maintained over 94% viability across a 7-day culture period. Further, histological sections obtained from the wounds 28 days after treatment showed a superior restoration of overall epidermal thickness and dermal collagen density	Pigs	Results indicated that bioprinting with amniotic fluid-derived (AFS) cells could be an effective treatment for large-scale wounds and burns. AFS cells were used in the bioink due to their high proliferation capacity, multipotency, immunomodulatory activity, and lack of significant immunogenicity	Fibrin in the bioink allowed for a transient scaffolding material with a safe degradation profile that readily attaches to and remodels prior to secreting its own extracellular matrix. The device is compact, light and can be operated with one hand	Cheng et al. (2020)
Portable handheld extrusion bioprinter	Aqueous 2-phase emulsion bioink composed of gelatin methacryloyl (GelMA) solution and PEO solution embedded with NIH/3T3 fibroblasts	Skin	The bioprinted porous GelMA hydrogel and control GelMA hydrogel had similar viability of NIH/3T3 fibroblasts. The porous GelMA hydrogels exhibited significantly faster cell proliferation than the control. Fibroblasts maintained high viability in porous hydrogels (>90%) even after performing compression cycles	NA	The handheld printer accurately filled in defects in porcine skin tissue. The porous GelMA construct allowed for high liquid and oxygen transport which is necessary for effective delivery of nutrients to cells. The high spreading and fast proliferation of fibroblasts in the porous bioscaffold demonstrates the potential for this technology in rapid wound healing	The inexpensive bioprinter included a motorized extrusion system, removable photocuring unit and portable battery. The two-phase emulsion bioink resulted in a porous hydrogel which allowed for liquid and oxygen transport, cellular proliferation and good elasticity	Ying et al. (2020)
Handheld bioprinter equipped with UV light source	Photopolymerizable nanoengineered bioink known as ‘muscle ink’	Muscle	C2C12 myoblast cultures in printed scaffolds had similar viability compared to tissue culture plate control after 3 days. Histological analysis of mice muscle showed that muscle and hydrogel were able to form a stable interface and that cells were able to infiltrate the hydrogel scaffold	Mice	Muscle ink adhered to skeletal muscle and gradually released vascular endothelial growth factor (VEGF) into surrounding tissue to help promote angiogenesis. Muscle ink constructs could be compressed up to 50% strain without failure. In a murine model, muscle ink has been shown to promote muscle recovery, reduce fibrosis, and increase anabolic response	The bioprinter allowed for fine-tuned continuous extrusion control, rapid exchange of bioink syringes, thermal insulation of syringes, and UV crosslinking. The designed bioink could release VEGF in a controlled manner over a period longer than 3 weeks	Quint et al. (2021)

## Bioprintable materials

**FIGURE 2:**
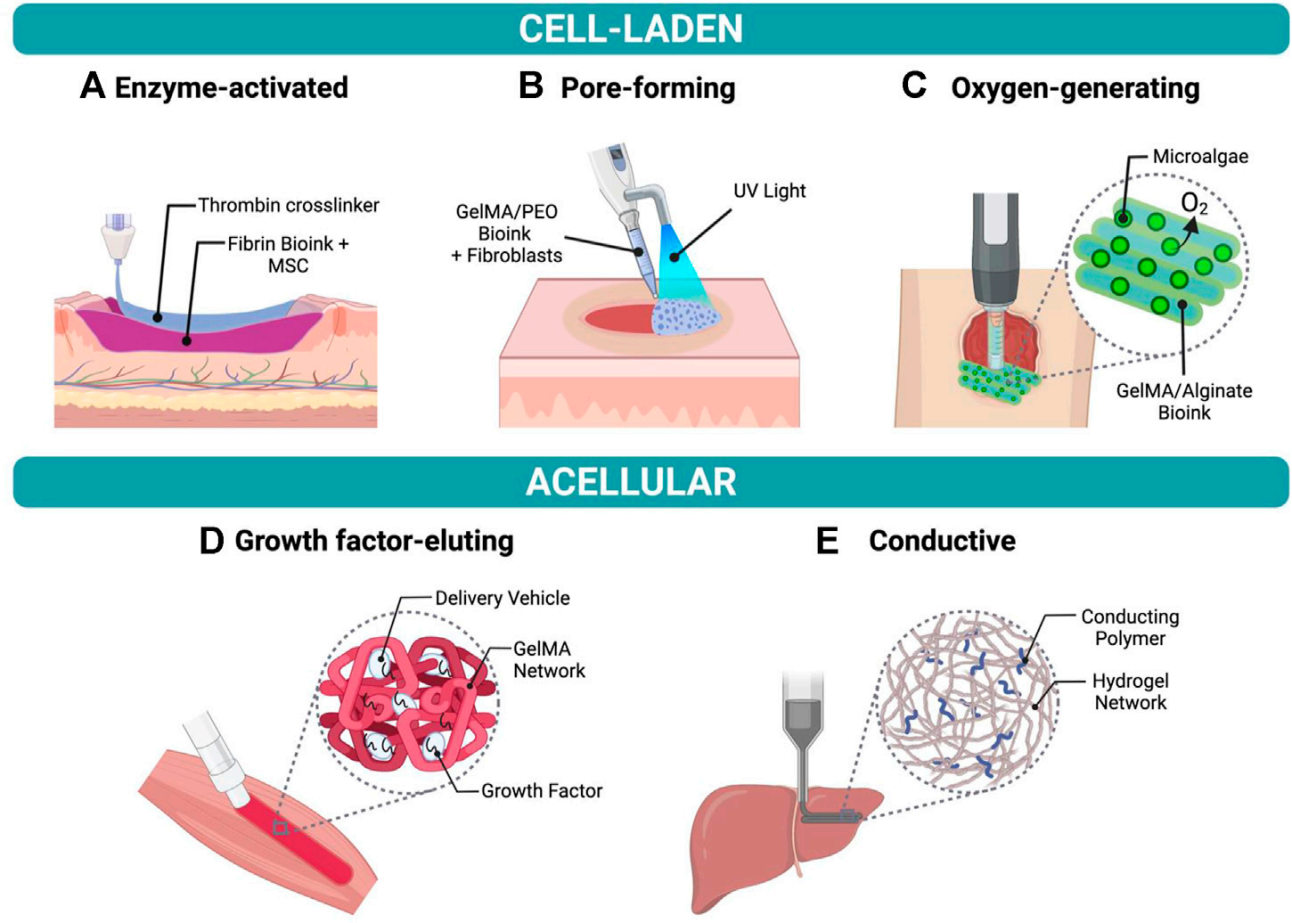
Examples of bioinks for *in situ* bioprinting **(A)** Fibrin-based bioink with mesenchymal stem cells (MSCs) crosslinked using thrombin to form a hydrogel in a skin wound bed. **(B)** Fibroblast-laden bioink composed of gelatin methacrylate (GelMA) and polyethylene oxide (PEO) photocrosslinked using UV light to create a porous scaffold. **(C)** GelMA and alginate bioink containing oxygen-producing microalgae to treat chronic wounds. **(D)** Growth factor-eluting bioink applied to muscle injury to promote functional muscle recovery. **(E)** Conductive hydrogel composed of hyaluronic acid and pluronic-F-127 printed onto liver.

The selection of biomaterials for *in situ* bioprinting is extremely important to ensure high printing resolution (<100 µm), fast *in situ* gelation, tissue regeneration and comparable mechanical properties between the printed architecture and the target tissue (Dias et al., 2020). As a result, a variety of bioprintable materials have been designed for *in situ* bioprinting onto different organs of interest, as shown in Figure 2.

### Bioinks

Bioinks are one of the most common biomaterials used in situ bioprinting. The dispersion of cells in bioinks helps promote cell proliferation and facilitate tissue formation after printing (Hospodiuk et al., 2017). Di Bella et al. (2018) used a gelatin methacrylate (GelMA) and hyaluronic acid (HA) methacrylate bioink with mesenchymal stem cells (MSCs) to treat cartilage injuries in sheep. The *in situ* printed bioscaffolds showed early signs of cartilage regeneration; however, weak adhesion of the printed material to host tissue prevented lateral integration. Hakimi et al. (2018) prepared fibrin-based bioinks and alginate-based bioinks to print multilayered sheets on murine and porcine skin wound models. The printed sheets remained firmly attached to the wound forming a hemostatic barrier immediately after application to the injured site. However, the biomaterial sheets did not significantly promote granulation tissue formation nor re-epithelization compared to control wounds. Recently, the same authors developed MSC-containing fibrin-HA bioinks to create skin precursor sheets directly onto porcine full-thickness burns (Cheng et al., 2020). The MSC-fibrin treated wounds exhibited reduced inflammation, scarring and contraction along with improved re-epithelialization. Although MSCs have been shown to enhance wound healing, these cells are relatively difficult to expand *in vitro* (Joo et al., 2012). In contrast, amniotic fluid-derived stem cells (AFS) are simpler to process and proliferate rapidly with a doubling time of 30–36 h (De Coppi et al., 2007). As a result, Skardal et al. (2012). employed AFS-laden fibrin bioinks in their bioprinter to treat large skin wounds in mice. The AFS-containing material similarly improved re-epithelialization compared to control MSC-containing material, and in fact, showed higher microvessel density and capillary diameters. Despite AFS-laden hydrogels promoting wound closure, migration of AFS cells into surrounding tissue was not observed. Potentially, the pore size of the fibrin-based hydrogel was not large enough to support AFS cell proliferation and spreading into the underlying tissue.

Ying et al. designed a tunable pore-forming GelMA/polyethylene oxide emulsion bioink containing NIH/3T3 fibroblasts for skin healing (Ying et al., 2020). Polyethylene oxide droplets were suspended in photocrosslinkable GelMA solution and subsequently removed after crosslinking GelMA to form a hydrogel matrix. The porous bioink-generated hydrogel facilitated liquid and oxygen transport, cellular proliferation, and exhibited good elasticity. The bioink has still not been tested *in vivo* thus tissue adhesion and toxicity due to free radicals generated during photocrosslinking must be assessed (Sharifi et al., 2021). Oxygen transport to tissues can also be achieved by incorporating peroxides or fluorocarbons into bioinks. However, these oxygen-generating compounds cannot sustain sufficient oxygen release over the entire healing process (Shiekh et al., 2020; Guan et al., 2021). To address this issue, Wang et al. incorporated living photosynthetic microalgae into their bioink to deliver a continuous supply of oxygen to the target tissue after *in situ* bioprinting (Wang et al., 2022). The microalgae-laden bioink enhanced chronic wound healing in mice after illuminating the bioprinted scaffold with LED light. Increasing oxygen availability in printed constructs *in situ* can induce tissue repair by alleviating local hypoxia, accelerating angiogenesis, and promoting extracellular matrix synthesis. Although microalgae-based scaffolds can provide sufficient oxygen to tissues under high light illumination (6,000 lux), oxygen generation significantly decreases when the light source is removed. The need to deliver continuous light to the printed microalgae construct as to avoid reduction in oxygen generation will be a real challenge when translating this technology to the clinic. Further, the light requirement of the microalgae limits the bioink’s use to repairing wounds on or near the skin’s surface.

The incorporation of xenogeneic or allogenic cells into bioinks allow the bioink production process to be potentially scaled up and automated. However, utilization of genetically different cells in bioinks carry the risk of immunological rejection. An alternative approach is to use autologous cells in bioinks as to avoid generating an immune response. Albanna et al. (2019) successfully printed autologous dermal fibroblasts and epidermal keratinocytes onto porcine skin wounds. The printed skin cells accelerated wound closure compared to control groups. Although this study demonstrated the promise of autologous cell-laden bioinks, few other studies have implemented these cells in their bioinks. The lack of research on patient-specific bioinks is likely due to limited tissue availability, cell morbidity at harvest site and time-consuming extraction and culturing processes (Diederichs et al., 2013). However, overcoming these shortcomings will be instrumental in delivering personalized therapies in the future.

### Biomaterial inks

Cell-laden bioinks present numerous advantages related to tissue repair and regeneration, yet high cell costs and lengthy culturing processes has resulted in a push towards acellular printable materials. In the field of biofabrication, these acellular materials are referred to as biomaterial inks (Groll et al., 2018). As a result, Ma et al. (2020) developed a cartilage repairing biomaterial ink which consisted of HAMA as well as acrylate-terminated 4-armed polyethylene glycol (PEG) to enhance the mechanical properties of the resultant photocrosslinked material. The purified HAMA and acrylate-terminated 4-Armed PEG were dissolved in phosphate-buffered saline (PBS) solution before bioprinting. This biomaterial ink proved to be successful as the cartilage defects were repaired in the hydrogel implantation group, and compared to the control group, the regenerated tissue exhibited a glossy and smooth appearance like that of native cartilage. Biomechanical properties of the regenerated cartilage were unable to mimic that of native cartilage despite resembling native cartilage microstructure. Urciuolo et al. synthesized 7-hydroxycoumarin-3-carboxylate (HCC) modified 4-arm PEG and gelatin biomaterial inks that could be finely tuned by varying laser power to achieve different biomechanical stiffnesses comparable to most native tissues (Urciuolo et al., 2020). Developed biomaterials were injected *in vivo* into skin, muscle, and brain and then crosslinked by two-photon cycloaddition forming a stable hydrogel within tissues. Hydrogels showed high biocompatibility but they did not demonstrate tissue regenerative properties unless stem cells were incorporated into the biomaterial ink.

Recently, Quint et al. (2021) created a growth-factor eluting biomaterial ink to promote muscle recovery. The biomaterial ink, referred to as muscle ink, formed a GelMA hydrogel *in situ* after photopolymerization, and then slowly released vascular endothelial growth factor (VEGF) to surrounding muscle tissues for more than 3 weeks. The sustained release of VEGF *in vivo* was accomplished by incorporating a nanoscale delivery vehicle known as laponite nanoclay along with VEGF into the biomaterial ink. Bioprinting of muscle ink in a murine model led to functional muscle recovery, reduced fibrosis, and increased anabolic response compared to untreated injured muscle. In another study, Zhou et al. (2021) used a conductive hydrogel biomaterial ink to print 3D structures onto live rat livers and post-mortem pig hearts. The advantage of using conductive biomaterial inks in bioprinting is that these materials have the potential to facilitate the propagation of electrical signals to cells; a process that is critical for cardiac and nerve regeneration (Min et al., 2018). Their conductive biomaterial ink composed of HA and pluronic-F-127 became a hydrogel at physiological temperatures due to the thermo-sensitive nature of the HA biomaterial ink (Jung et al., 2017). Moreover, *ex vivo* and *in situ* bioprinting of the conductive biomaterial ink demonstrated good adhesion of the material to target tissue. However, this study was only a proof-of-concept and the authors still need to test the regenerative properties of their employed biomaterial ink *in vivo* (Min et al., 2018).

Keriquel et al. focused on repairing calvarial bone defects using nano-hydroxyapatite (n-HA) as their biomaterial ink (Keriquel et al., 2010). This material was shown to be biocompatible with osteoblastic cells and caused no inflammation *in vivo*. One month after bioprinting n-HA biomaterial ink in mice, newly formed mature and immature bones and n-HA aggregates were found inside macrophages, whereas no bone repair was seen at control sites. However, x-ray micro-tomography revealed that bone repair was not significantly higher in the n-HA-treated group compared to the control group. Further, mechanical strength testing was not performed on calvarial defects to assess long bone repair. An ongoing problem for bioinks designed for hard tissue applications is matching the mechanical strength of human bone while maintaining sufficient porosity. Specifically, bioinks based solely on hydroxyapatite can achieve high porosity but are unable to replicate the mechanical properties of bone tissue (Abdul Halim et al., 2021). The development of composite materials with high fracture toughness should increase the growth of *in situ* bioprinting in hard tissue engineering.

## Bedside mounted bioprinting

One of the most common bioprinters are bedside mounted bioprinters that deposit bioinks or biomaterial inks onto injured sites using computer-generated designs (Singh et al., 2020). Bedside mounted bioprinters take advantage of medical imaging and computer-aided design software to create 3D wound models that are used to program the final designs employed by bioprinters. Li et al. (2017) used a handheld 3D scanner to develop 3D models of rabbit osteochondral defects to be used for *in situ* bioprinting. The portable 3D scanner generated high-resolution scans of the osteochondral defects in minutes. However, this 3D scanner would not be able to model internal organs unless the organs were made visible to the scanner during a surgical procedure, thus in effect, limiting its applicability. Magnetic resonance imaging, another imaging technique often used in 3D modeling, can be used to determine the structure of target organs inside the body. The main drawbacks of this technique include its high cost, and poor imaging capability of hard tissues (Mastrogiacomo et al., 2019). Zhou et al. (2021) implemented computed tomography (CT) to reconstruct the surface of a rat liver pre-operation. Although CT imaging can be used to visualize soft tissues such as liver, CT is less effective compared to other techniques at differentiating soft tissues which can lead to less accurate modeling (Wood et al., 2018). Further, this technique requires the use of harmful radiation, which poses a risk to human health (Chromy and Zalud, 2014). As a result, the type of imaging technique used in 3D modeling should be dependent on the printing location and target tissue properties. For example, a 3D scanner could be the ideal method for developing a skin wound model, but CT could be the best option for modelling cortical bone defects. In some cases, a combination of different imaging techniques may be desired to develop a more accurate model for *in situ* bioprinting. After developing a 3D model of a wound site, the precise geometry of the to-be printed construct can be designed using computer-aided design (CAD) software (Pakhomova et al., 2020). The generated CAD files can then be processed by computer-assisted bioprinters as to 3D print the desired structures onto or into damaged tissues. The printer head of a bedside mounted bioprinter is designed to move along the x, y, and *z* axes in 3D space to precisely deliver bioink according to CAD file specifications. Although bedside mounted bioprinters function in similar ways, some key differences exist with relation to their delivery method, spatial flexibility, printing accuracy, and special features.

Skardal et al. (2021) developed a bioprinter with three-axis movement capability and multiple sets of pressure driven nozzles for delivering bioinks. The bioprinter was designed to deposit alternating layers of hydrogel solution and crosslinker solution onto skin wounds and its efficacy was tested in mice. Similarly, a team from the University of Iowa used pressure-driven nozzles in the design of their bioprinter but rather than employing standard bioprinting nozzles, they used a co-axial nozzle to deliver hydrogel and crosslinker solutions simultaneously. Furthermore, the developed bioprinter had a second dispensing arm which could be used to deliver cell spheroids concurrently to the wound site thus reducing total printing time. Although the secondary dispensing arm increased printing speeds, the multi-arm bioprinter suffered from limited spatial flexibility which is problematic when printing *in situ* onto non-planar defect surfaces (Ozbolat et al., 2014).

Keriquel et al. (2010) built a bioprinter with a sophisticated five-axis positioning system to allow for easier manipulation of specimen positioning. The bioprinter was equipped with an infrared pulsed laser which was used to generate biomaterial ink microdroplets from the ribbon surface for bioprinting. Despite the high degree of precision associated with laser-assisted bioprinting, the authors noted that the precision and accuracy could be further improved by incorporating medical robots into bioprinters. Ma et al. (2020) integrated a 6-degree-of-freedom (6-DOF) robot and fast calibration tool with their bioprinter to improve *in situ* printing accuracy. The integrated 6-DOF robot also allowed their bioprinter to operate within a larger workspace that would be favorable for performing a variety of surgical procedures in operating rooms. The biomaterial ink deposited by their bioprinter was crosslinked post printing using ultraviolet (UV) light. High-energy UV light can induce photochemical damage in tissues which prompted researchers to investigate longer wavelength light as an alternative for *in vivo* applications (Zheng et al., 2021). For instance, Urciuolo et al. (2020) took advantage of low energy near infrared laser light to crosslink photoactive biopolymers using their intravital 3D bioprinting technology. The authors injected bioinks into various tissues of live mice and then used a commonly available multiphoton microscope to create predefined geometries within these tissues. However, intravital bioprinting was limited to superficial anatomical sites that could be exposed to the light source. Ferromagnetic soft catheter robots (FSCRs) are not restricted by tissue penetrability of light and can print constructs on internal organs well beneath the skin’s surface (up to 150 mm printing depth) (Zhou et al., 2021). FSCRs can be inserted through a small incision on the skin’s surface and their tip can be controlled remotely using magnetic actuation to print over large surfaces. While the use of FSCRs in bioprinting present many advantages, the technology is still in its infancy and significant work is needed to show its effectiveness in printing more complex structures in large animal models.

In general, bedside mounted bioprinters can print various geometries *in situ* while achieving high printing speed and accuracy. Some key challenges bedside mounted bioprinters may face when the technology is to be translated to the clinic will be the need for medical imaging and trained personnel to develop 3D wound models (Saini et al., 2021). The high cost and complexity of these bioprinters may also reduce its appeal for healthcare workers. Improvements made to the user interface of these bioprinters should reduce the amount of training required for bioprinter operation. Further, the development of portable and low-cost medical imaging modalities such as mobile CT and 3D scanners should help bedside mounted bioprinters become more prevalent in healthcare environments.

## Hand-held bioprinting

The other type of printing technique used *in situ* 3D bioprinting are hand-held printers. Unlike bedside mounted printers which are generally built in such a way that it fits around the subject, a hand-held printer, like the name entails, can be brought to the subject. Consequently, they solve a major problem encountered with bedside mounted printers as they can generally be used regardless of the size of the object. Further, handheld bioprinters avoid the use of medical imaging and CAD wound modelling, thus reducing the total cost, complexity and preparation time associated with the bioprinting process. The manual operation of the medical device provides users with an increased amount of flexibility when completing surgeries and amendments to desired structures, increasing surgical dexterity. Additionally, the small nature of the handheld device allows for it to be portable in and out of surgery, as well as easily sterilized.

One of the most well-known hand-held bioprinting devices is the Biopen (O’Connell et al., 2016). This device works in such a way that the object being printed is manually and directly written into the subject. This method of bioprinting allows for design modifications to occur in real-time while the surgeon adapts to slight changes in the tissue microenvironment. In effect, the final construct geometry is entirely dependent on the user’s discretion. The original Biopen permitted stable extrusion of bioinks but could not achieve high resolution without inducing shear stress cell damage. As a result, Di Bella et al. employed an upgraded version of the Biopen to repair cartilage defects in sheep. The upgraded Biopen had a multi-inlet extruder nozzle which allowed the authors to print cell-laden materials enclosed in a protective biomaterial shell to limit shear stress-related cell damage. In addition, the handheld printer included a UV light source to facilitate photocrosslinking of bioinks, two chambers to hold core/shell bioinks, and a motorized control system.

Quint et al. (2021) designed a handheld bioprinter to deliver their unique eluting biomaterial ink to skeletal muscle injuries. Their bioprinter was similarly equipped with an embedded UV light source and could print continuous and uniform fibers onto damaged muscle tissues. In contrast to the Biopen, this device was battery-powered and had a micro-USB port located at the back of the device to enable fast charging. Further, the bioprinter design enabled thermal insulation of incorporated bioinks; a special feature particularly beneficial for thermosensitive bioinks. Recently, Ying et al. (2020) engineered a low-cost, battery-powered printer to deposit bioinks onto skin wounds. The entire bioprinter could be built for ≈$100 USD with all its software and hardware components made completely open source. Bioink syringes could be exchanged quickly due to a built-in function in the device which allowed the bioprinter’s syringe push plate to be rapidly retracted to its fully extended position. Further, their handheld extruder contained a detachable UV crosslinking system which could be replaced by a lower-wavelength light source to limit photo-induced tissue damage.

As an alternative to photocuring-based handheld printers, Hakimi et al. (2018) built a printer that could facilitate crosslinking of enzyme-activated and ionic-activated materials. Their instrument included a pair of actively driven rollers to control translation speed over the wound bed during biomaterial deposition. Biomaterial and crosslinking solutions could be loaded individually into their device and then extruded concurrently through a microfluidic cartridge resulting in a consistent tissue sheet covering the injured anatomical region. The microfluidic cartridge was designed to allow the crosslinker solution to be delivered directly above the bioink layer to initiate rapid gelation. Printed sheet dimensions could be controlled by altering the microfluidic cartridge size, bioink flowrate, and crosslinker flowrate according to the user’s preferences. Recently, Cheng et al. (2020). made some improvements to the bioprinter. The authors incorporated a heat transfer network into the instrument to control the temperature of the delivered bioink. In effect, the instrument provided users with the option to finetune bioink temperature to optimize the materials bioprintability (Janmaleki et al., 2020). Further, the microfluidic printhead of their device was imparted with two rotational degrees of freedom to ensure accurate and continuous printing on inclined surfaces. The original bioprinter design by Hakimi et al. did not permit multi-axis rotation of the microfluidic printhead and thus could only print high-resolution sheets on modestly inclined wound surfaces (Hakimi et al., 2018).

In general, the deposition of bioinks as sheets rather than lines increases printing speeds but limits the complexity of the constructs that can be printed. The greater control associated with line-forming bioprinters allows for the reconstruction of a greater variety of anatomical structures. As a result, sheet-generating bioprinters have mainly been used to treat large-area skin wounds which have a reasonably smooth topology whereas bioprinters such as the Biopen have been used to repair various irregular defects (Di Bella et al., 2018; Onofrillo et al., 2018).

Handheld bioprinters have the potential to be adopted by the clinic without significant alterations to their design. These printers are easy-to-use and require minimal operator training for surgeons (Hakimi et al., 2018). Clinical success of handheld printers will depend primarily on the advancement of bioprintable materials. For one, tissue damage during *in situ* gelation is currently an issue for many bioprintable materials. Further, it is difficult to produce large quantities of bioinks for bioprinting since large scale cell production is not yet widespread (Kami et al., 2013). However, the development of low energy photocrosslinkable bioinks and crosslinker-free bioinks should significantly reduce toxicity risk, and in effect increase the demand for handheld bioprinters. Another obstacle that must be overcome for handheld bioprinters to be accepted by the healthcare community is the need for an ergonomic printer design that is satisfactory for different users; printer designs should account for varying hand sizes, and whether the user is right-handed or left-handed. Also, the printer’s weight, positioning of controls, balance, and materials should be modified to maximize user comfort (Pazhouhnia et al., 2022). These modifications should be straightforward for engineers to implement into their designs. For instance, printers could avoid problems with surgeon hand size by utilizing detachable handles.

## Bioprinting techniques in minimally invasive surgery

Bedside-mounted and handheld bioprinters have both shown promise in minimally invasive surgery. The portability and small size of handheld bioprinters make them a great option for printing 3D structures within confined areas (Di Bella et al., 2018). Most handheld printers have small-diameter nozzles which can be inserted into minor incisions in the skin to print on the surface of internal organs. The surgeon can guide the nozzle tip to the defect site and initiate bioink deposition to restore the original tissue structure. Handheld printers which are equipped with a microfluidic printhead are less ideal for minimally invasive surgery due to the larger size of the printhead (Hakimi et al., 2018).

Although nozzle-based handheld instruments have great potential for minimally invasive bioprinting, there have been only a few attempts to print constructs *in situ* using this approach. The complexities associated with printing materials in a non-invasive manner is likely the main reason for why minimally invasive bioprinting has not progressed significantly. Despite these challenges, Vimex is currently developing and manufacturing a handheld bioprinter specifically designed for minimally invasive bioprinting onto cartilage injuries (Gapiński et al., 2020). Their arthroscopic printing tool can be inserted deeper under the skin’s surface than other devices to repair chondral defects thanks to its long narrow neck. Surgeons would employ this handheld extruder in tandem with a secondary arthroscopic tool equipped with a video camera to visualize *in situ* chondral defects (Krzysztof et al., 2019). The company showed proof-of-concept of their minimally invasive approach using a knee phantom. The authors noted, however, that upon entering the operation area with their extruder, the extrusion tip could become blocked or partially blocked by biological debris inside the cavity.

The traditional design of bedside-mounted bioprinters had to be reimagined for minimally invasive surgery. Unlike manually driven handheld devices, the printheads of conventional bioprinters are not capable of rotational movement. The lack of rotational control of conventional bioprinters restrict their access within small apertures during minimally invasive surgery. Therefore, researchers developed robotic-assisted bioprinters with enhanced range of motion to widen the printing range of bioprinters in compact areas (Lipskas et al., 2019; Ma et al., 2020). These bioprinters updated with high degree of freedom robots have been used *in vivo* to repair cartilage defects in rabbits but they have not yet been tested through a minimally invasive approach. Ferromagnetic soft catheter robots (FSCRs) have also shown potential in minimally invasive bioprinting. As opposed to rigid nozzles used in conventional bioprinting, FSCR nozzles are soft and can bend in multiple directions to navigate diverse biological environments due to magnetic actuation (Zhou et al., 2021). The ability of FSCRs to change their curvature can be exploited to enter small apertures as a linear rod-like structure and repair defects out of line with the initial entry point.

Bioprinters utilizing near-infrared (NIR) light have been able to print complex shapes beneath the skin’s surface without creating an open wound (Urciuolo et al., 2020). This type of non-invasive bioprinting is realized by subcutaneously injecting photopolymerizable bioink into the desired anatomical site and subsequently crosslinking the bioink by applying highly controlled NIR light. Chen et al. printed an ear-shaped construct beneath the skin *in vivo* by modulating NIR using a digital micromirror device (Chen et al., 2020). Urciuolo et al. (2020) printed constructs in skin, muscle and brain using a multiphoton NIR laser-scanning microscope. Both these techniques led to fast printing and high-resolution constructs. The main limitation of NIR-dependent techniques relates to its maximum fabrication depth which is only a few millimeters beneath the skin’s surface. Therefore, further work will need to be done to improve light penetration in tissues before this non-invasive bioprinting approach can be applied for deep tissue repair.

Another non-invasive bioprinting approach was proposed by Zhao and Xu. (2020) to treat gastric wounds. The authors envisioned delivering bioinks to gastric wall injuries using a micro bioprinting platform installed on an endoscope. Therefore, the authors constructed a miniature delta robot which could fold into a compact state when traveling through the body and unfold once it reached the wound site. To test the efficacy of their design, the miniaturized robot was attached to an endoscope and connected to a syringe filled with bioink by a long polytetrafluoroethylene tube. Then, the novel bioprinting system was used to print a 2-layer lattice structure *in vitro*. In addition to repairing gastric wound sites, the micro bioprinting system may find applications in repairing other anatomical tissues. Currently, the micro bioprinter has not been tested *in vivo* because it is still a bit too large for practical endoscopy (Patel et al., 2015). Although bioprinting systems such as the one proposed here must be further miniaturized to be effective in clinical applications, micro bioprinters present a promising way forward to repair damaged or diseased tissue without interfering with normal physiological processes.

## Outlook

The last few decades have brought us closer to what in the 50 s was considered as science fiction. For the first time in modern society, we are a step closer to see tissues repaired in real time with an unprecedented precision. However, at the same time, we are reminded of the complexity that mimicking tissues entails. As the field of 3D bioprinting keeps growing, there is also a need to aggressively invest in clinical use of bioprinting beyond the archetypical creation of tissues to be post implanted. Rapid and precision quality repair using *in situ* bioprinting needs probably two more decades to fully mature technologically. However, relative to other technologies for biomedical application, technological adoption seems not to be a significative barrier probably due to the fact *in situ* bioprinting was born of the urgent need to better repair tissues and organs.
